# First-Line Maintenance Therapy Patterns in BRCA Wild-Type, HRD-Negative or Biomarker-Unknown Advanced Epithelial Ovarian Cancer: A Five-Country European Real-World Study by APLUSA

**DOI:** 10.3390/cancers18162682

**Published:** 2026-08-19

**Authors:** Mario Uccello, Stergios Boussios, Christine Maï, Hilary Worton, Arthur Bazire, Bertrand de Buhren

**Affiliations:** 1 Department of Oncology, Royal United Hospitals Bath NHS Foundation Trust, Bath BA1 3NG, UK; 2 Department of Oncology, University of Ioannina, 45500 Ioannina, Greece; stergiosboussios@uoi.gr; 3 Clinical Research, APLUSA, 69003 Lyon, France; 4 Clinical Research, APLUSA UK, London SE1 8EN, UK; h.worton@aplusaresearch.com

**Keywords:** advanced epithelial ovarian cancer, first-line maintenance therapy, BRCA wild-type, homologous recombination deficiency, real-world evidence

## Abstract

**Background/Objectives:** First-line maintenance is established in advanced epithelial ovarian cancer (AEOC), but management remains uncertain for patients without a BRCA mutation or homologous recombination deficiency (HRD)-positive disease. We assessed real-world use of bevacizumab, poly(ADP-ribose) polymerase (PARP) inhibitors and active surveillance in France (FR), Germany (DE), Italy (IT), Spain (ES) and the United Kingdom (UK). **Methods:** Anonymised physician-reported charts were analysed from a retrospective, non-interventional online survey conducted by AplusA Healthcare Marketing Research. Eligible patients had stage III–IV AEOC, no known BRCA-mutated or HRD-positive disease, no progression after first-line platinum chemotherapy, and started maintenance therapy or active surveillance between November 2024 and March 2026. Treatment allocation was compared by chi-square testing. Multinomial logistic regression assessed whether country remained associated with strategy after adjustment for clinical and disease characteristics. **Results:** Among 3293 patients, 1295 (39.3%) received bevacizumab, 1087 (33.0%) niraparib, 158 (4.8%) rucaparib, 477 (14.5%) active surveillance and 276 (8.4%) other therapy. Treatment allocation differed by country (χ^2^ = 725.4, df = 16, *p* < 0.001). Bevacizumab was most frequent in FR and least frequent in the UK; niraparib was most frequent in ES and the UK; active surveillance was most common in the UK and IT. In the adjusted model, country was the strongest factor associated with strategy (likelihood-ratio χ^2^ = 550.1, df = 8, *p* < 0.001). **Conclusions:** First-line maintenance practice in AEOC without an actionable BRCA/HRD biomarker varies substantially across European countries. These differences may partly reflect national access, reimbursement, clinical practice and other unmeasured country-level factors. Outcome-based real-world studies are needed to clarify the best strategy.

## Simple Summary

Many women with advanced epithelial ovarian cancer do not have a biomarker to guide treatment decisions after initial chemotherapy. In this situation, doctors may choose different post-chemotherapy strategies depending on patient factors, local practice and national access to medicines. This study looked at real-world treatment choices in France, Germany, Italy, Spain and the United Kingdom for patients without a known biomarker result supporting a specific targeted maintenance approach. The aim was to describe how often different strategies were used and whether choices differed between countries after accounting for patient and disease characteristics. The findings show substantial variation between countries, which may partly reflect differences in national access, reimbursement and clinical practice. These results may help guide future outcome-based studies comparing post-chemotherapy strategies in patients without a biomarker to direct maintenance treatment.

## 1. Introduction

Maintenance therapy has become an integral component of first-line treatment for advanced epithelial ovarian cancer (AEOC) after response to platinum-based chemotherapy [1,2]. The treatment landscape has evolved substantially with the introduction of bevacizumab and poly(ADP-ribose) polymerase (PARP) inhibitors, which have improved progression-free survival (PFS) in selected patient populations [3,4,5,6]. However, effects on overall survival (OS) have been less consistent and may be influenced by subsequent therapies and treatment crossover [7,8,9,10,11]. Treatment selection in AEOC is increasingly shaped by molecular stratification. Patients with BRCA-mutated or homologous recombination deficiency (HRD)-positive disease derive the greatest benefit from PARP-inhibitor-based maintenance, and these groups have become central to contemporary treatment algorithms. By contrast, the optimal approach for patients with BRCA wild-type and HRD-negative disease, or those in whom HRD status is unknown, remains less clearly defined. This population is clinically important because it represents a substantial proportion of patients encountered in routine practice, yet the magnitude of benefit from PARP inhibition is generally smaller than that observed in biomarker-selected disease [9,10,12]. Several first-line maintenance strategies may be considered in this setting. Bevacizumab, an anti-vascular endothelial growth factor (VEGF) agent, remains an established option, particularly for patients who receive bevacizumab with first-line chemotherapy and continue treatment as maintenance [13]. Niraparib and rucaparib have both demonstrated benefit as first-line maintenance monotherapy after response to platinum-based chemotherapy, including in populations not restricted by BRCA or HRD status [14,15]. Conversely, active surveillance remains a reasonable strategy for selected patients, particularly when the expected benefit is modest, toxicity is a concern, or treatment escalation is not considered appropriate [9,12,15]. Despite the availability of these options, real-world prescribing patterns in biomarker-negative or -unknown AEOC remain incompletely described. Existing studies have largely focused on broad first-line maintenance uptake, PARP inhibitor use, or outcomes associated with individual agents, rather than on country-level variation within BRCA wild-type, HRD-negative or biomarker-unknown populations [16,17]. Such variation is clinically relevant because maintenance treatment allocation may be associated not only with interpretation of trial evidence, but also with regulatory approvals, reimbursement criteria, biomarker testing infrastructure, clinician preference, and national treatment pathways [12,16,18]. In this European real-world study using physician-reported patient chart data, we evaluated first-line maintenance therapy patterns among patients with stage III–IV BRCA wild-type, HRD-negative or HRD-unknown AEOC across five European countries: France (FR), Germany (DE), Italy (IT), Spain (ES), and the United Kingdom (UK). We aimed to describe the distribution of maintenance strategies, determine whether treatment allocation differed significantly between countries, and assess whether country remained associated with maintenance allocation after adjustment for baseline clinical and disease characteristics.

## 2. Patients and Methods

### 2.1. Study Design and Data Source

This was a retrospective, non-interventional, physician-reported real-world study based on anonymised patient record forms completed by treating physicians through a self-administered online questionnaire conducted by APLUSA, a global healthcare market research company. Data were reported by participating physicians using information available in the medical records of patients under their care and were not independently abstracted by external chart reviewers. Physicians practising in FR, DE, IT, ES, and the UK were recruited through local agencies and screened using predefined eligibility criteria. Eligible participants were medical oncologists, oncologists, gynaecologic oncologists, or onco-haematologists with 2–35 years of professional experience, with at least 50% of their working time devoted to patient care, personal responsibility for prescribing or initiating treatment for patients with ovarian cancer, and a caseload of at least four patients with high-grade ovarian cancer. Information on practice type and geographic region was also collected. In each country, around 30 physicians were recruited per month, corresponding to up to 90 unique physicians participating in each quarter. Physicians could be recontacted in subsequent quarters, but no physician was permitted to participate more than once within the same quarter, and a minimum interval of two months was maintained between participations. Participating physicians were instructed to complete anonymised patient record forms for up to four of their most recently seen eligible AEOC patients under their care who had been seen within the preceding three months. The source database included patients across biomarker-defined subgroups, including biomarker-positive, biomarker-negative, and biomarker unknown/untested disease. For the present analysis, the study population was restricted to patients without a known BRCA mutation or HRD-positive result and comprised three distinct categories: (i) BRCA-wild-type/HRD-negative disease; (ii) BRCA-wild-type disease with HRD unknown or untested; and (iii) biomarker-unknown or untested disease not classified as BRCA-mutated or HRD-positive. These categories were not considered biologically equivalent; biomarker subgroup was therefore retained as a categorical variable in the descriptive and adjusted analyses. The categories were included within the same overarching cohort because none of the patients had a known positive BRCA/HRD result supporting a biomarker-selected maintenance treatment pathway. Eligible patients had initiated first-line maintenance therapy or active surveillance between November 2024 and March 2026.

### 2.2. Study Population

Eligible patients were adults with International Federation of Gynecology and Obstetrics (FIGO) stage III–IV AEOC who had received first-line platinum-based chemotherapy, with or without bevacizumab. Patients were eligible for inclusion in the present analysis if they had no evidence of disease progression after induction treatment and had entered the post-chemotherapy maintenance or surveillance phase. Surgical status was recorded as no debulking surgery, primary debulking surgery, or interval debulking surgery. For patients who underwent debulking surgery, residual disease status was recorded as no residual disease, optimal cytoreduction, or suboptimal cytoreduction. During the survey, response to induction therapy was recorded as complete response, partial response, stable disease or progressive disease. However, patients with disease progression after induction treatment were considered ineligible for maintenance therapy and were excluded from the present analysis. Patients were assigned to countries according to the location of the treating oncologist.

### 2.3. Variables and Objectives

The main variable of interest was first-line maintenance strategy after completion of platinum-based chemotherapy. Treatment categories were bevacizumab, niraparib, rucaparib, active surveillance, and other maintenance therapy. Patients who did not receive an active maintenance drug were classified as undergoing active surveillance. Available baseline and treatment-related variables included age category, Eastern Cooperative Oncology Group (ECOG) performance status, FIGO stage, histology, country, biomarker subgroup, response to induction therapy, surgical status, residual disease status after debulking surgery, and use of bevacizumab during first-line induction treatment. The primary objective was to describe the distribution of first-line maintenance strategies in the study cohort and by country. Secondary objectives were to assess whether maintenance allocation differed significantly between countries and, in an exploratory adjusted analysis, whether country remained associated with maintenance strategy after accounting for baseline clinical and disease characteristics.

### 2.4. Statistical Analysis

Categorical variables were summarised as frequencies and percentages. The distribution of first-line maintenance strategies was described in the overall cohort and separately for each country. Differences in maintenance allocation between countries were assessed using the chi-square test of independence. Adjusted standardised residuals were calculated to identify maintenance strategies with observed frequencies higher or lower than expected within each country under the null hypothesis of no association between country and maintenance strategy. Positive residuals indicated higher-than-expected use, whereas negative residuals indicated lower-than-expected use.

For the exploratory adjusted analysis, patients receiving treatment classified as other maintenance therapy were excluded. Maintenance strategy was grouped into three clinically relevant categories: bevacizumab maintenance, PARP inhibitor maintenance, and active surveillance. PARP inhibitor maintenance included niraparib and rucaparib. A multinomial logistic regression model was used with maintenance strategy as the dependent variable and country as the principal variable of interest. The model adjusted for age, FIGO stage, ECOG performance status, histology, biomarker subgroup, response to induction therapy, and surgical outcome. Use of bevacizumab during induction treatment was not included in the primary adjusted model, because it forms part of the treatment pathway leading to maintenance selection rather than a neutral baseline characteristic. Likelihood-ratio tests were used to compare the full multinomial logistic regression model with reduced models omitting each variable in turn. A sensitivity analysis repeated the adjusted model in patients with confirmed BRCA wild-type/HRD-negative disease. All statistical analyses were performed using R version 4.4.2 (R Foundation for Statistical Computing, Vienna, Austria) through RStudio version 2026.04.0+526 (Posit Software, PBC, Boston, MA, USA). Statistical tests were two-sided, and a *p* value of less than 0.05 was considered to indicate statistical significance. Given the descriptive nature of the analysis, residual findings were interpreted alongside absolute patient numbers and country-specific treatment proportions.

## 3. Results

### 3.1. Patient Population and Baseline Characteristics

The analysis included 3293 BRCA wild-type/HRD-negative or biomarker unknown/untested patients with FIGO stage III–IV AEOC who initiated first-line maintenance therapy or active surveillance between November 2024 and March 2026. Patients were treated in FR (*n* = 751), DE (*n* = 589), IT (*n* = 633), ES (*n* = 618), and the UK (*n* = 702). Baseline characteristics according to country are shown in Table 1. Most patients had BRCA wild-type/HRD-negative disease (2951 of 3293; 89.6%); 163 patients (4.9%) had BRCA wild-type disease with HRD unknown/untested status, and 179 (5.4%) had biomarker unknown/untested disease. Age was recorded in prespecified categories: 30 patients (0.9%) were younger than 40 years, 676 (20.5%) were aged 40–59 years, 1508 (45.8%) were aged 60–69 years, 931 (28.3%) were aged 70–79 years, and 148 (4.5%) were 80 years or older. High-grade serous histology was recorded in 2635 patients (80.0%). Overall, 1450 patients (44.0%) had FIGO stage III disease and 1843 (56.0%) had stage IV disease. ECOG performance status was 0 in 636 patients (19.3%), 1 in 2141 (65.0%), and 2 or higher in 516 (15.7%). Response to induction therapy was complete response in 1548 patients (47.0%), partial response in 1679 (51.0%), and stable disease in 66 (2.0%). Bevacizumab was used during induction treatment in 1189 patients (36.1%). In total, 2148 patients (65.2%) underwent debulking surgery. Surgical outcome was recorded as no debulking surgery in 1145 patients (34.8%), surgery with no residual disease in 942 (28.6%), surgery with optimal cytoreduction in 864 (26.2%), and surgery with suboptimal cytoreduction in 342 (10.4%). Baseline characteristics varied across countries, particularly with respect to FIGO stage, histology, use of bevacizumab during induction treatment, and surgical outcome.

### 3.2. First-Line Maintenance Treatment Allocation

**Figure 1:**
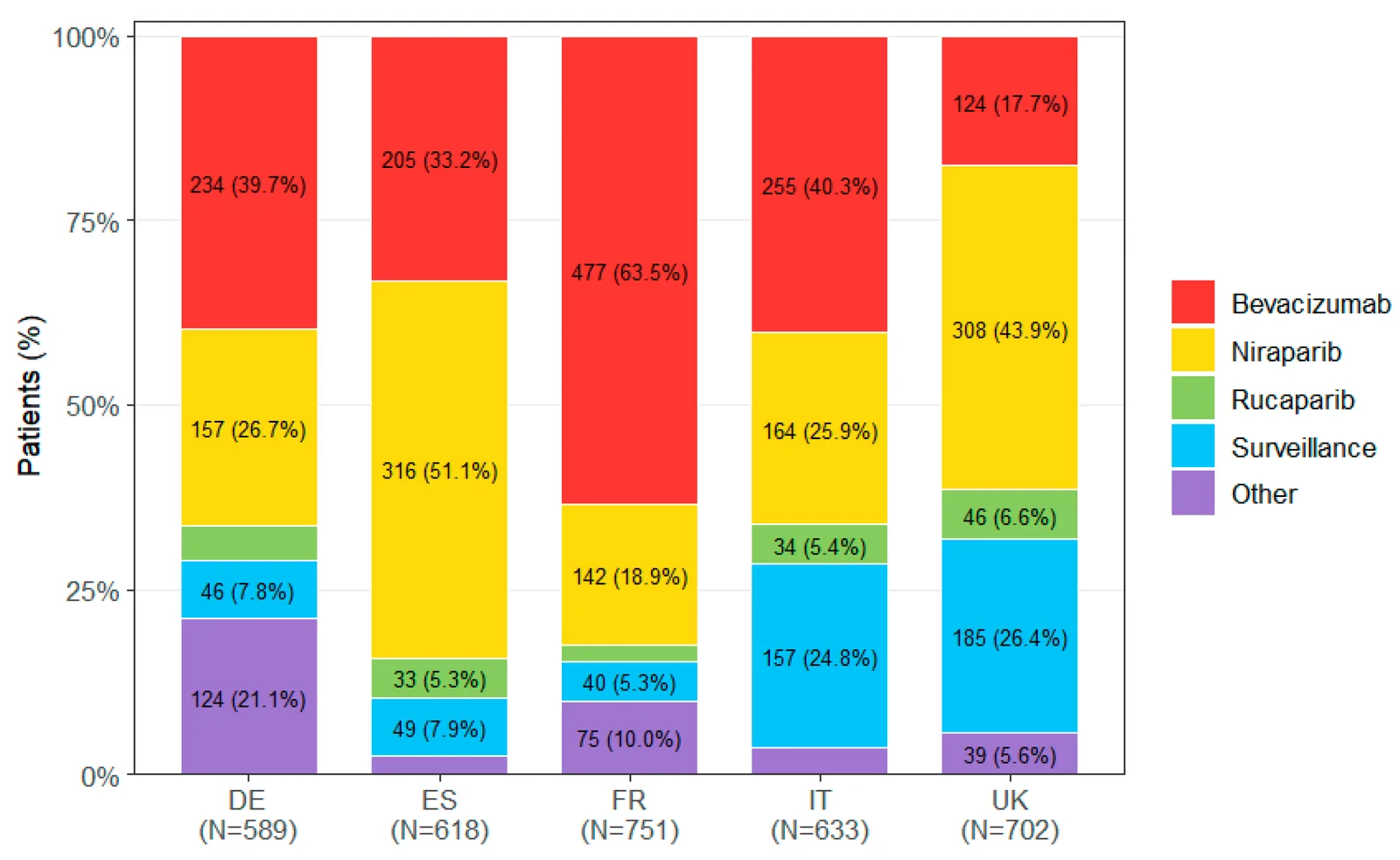
First-line maintenance treatment allocation by country. Bars show the percentage distribution of first-line maintenance strategies within each country. Values shown within bars are *n* (%). Small segments not labelled in the figure are as follows: DE, rucaparib 28 (4.8%); ES, other 15 (2.4%); FR, rucaparib 17 (2.3%); IT, other 23 (3.6%). Abbreviations: DE, Germany; ES, Spain; FR, France; IT, Italy; and UK, United Kingdom.

Figure 1 shows the distribution of first-line maintenance strategies by country. In the overall cohort, bevacizumab was used in 1295 patients (39.3%), niraparib in 1087 (33.0%), rucaparib in 158 (4.8%), active surveillance in 477 (14.5%), and other maintenance therapy in 276 (8.4%).

The distribution of maintenance strategies differed significantly by country (χ^2^ = 725.4, df = 16, *p* < 0.001). Bevacizumab use was highest in FR (*n* = 477; 63.5%) and lowest in the UK (*n* = 124; 17.7%), whereas niraparib use was highest in ES (*n* = 316; 51.1%) and the UK (*n* = 308; 43.9%) and lowest in FR (*n* = 142; 18.9%). Active surveillance was most frequent in the UK (*n* = 185; 26.4%) and IT (*n* = 157; 24.8%). Rucaparib use was infrequent across all countries. Table 2 shows the standardised residuals identifying the maintenance strategies most over- or under-represented in each country.

### 3.3. Multinomial Logistic Regression Analysis

A multinomial logistic regression model was used to assess whether country remained associated with first-line maintenance strategy after adjustment for baseline clinical characteristics. Patients receiving other maintenance therapy were excluded from this analysis; the model therefore included 3017 patients and compared three maintenance strategies: bevacizumab, PARP inhibitor maintenance, and active surveillance. Likelihood-ratio (LR) tests from the multinomial logistic regression model are shown in Table 3. Country was the strongest factor associated with maintenance strategy, with the largest reduction in model fit when removed from the full model (LR χ^2^ = 550.1, df = 8, *p* < 0.001). Surgical outcome, age, ECOG performance status, response to induction therapy, and histology were also associated with maintenance strategy, whereas biomarker subgroup and FIGO stage were not. A sensitivity analysis restricted to patients with confirmed BRCA wild-type/HRD-negative disease (*n* = 2734) produced findings consistent with the primary analysis. Country continued to show the strongest association with maintenance strategy (LR χ^2^ = 555.2, df = 8, *p* < 0.001), while the significance pattern of the remaining covariates was unchanged. Full multinomial logistic regression model estimates, including regression coefficients, odds ratios, 95% confidence intervals, and *p* values, are provided in Table S1.

## 4. Discussion

In this five-country European real-world study of patients with stage III–IV AEOC with BRCA-wild-type/HRD-negative or unknown biomarker status, first-line maintenance treatment allocation differed substantially between countries. This analysis extends a preliminary ASCO 2026 report of 977 patients treated between November 2024 and March 2025, in which similar country-level variation in first-line maintenance strategy was observed [19]. The present study includes 3293 patients treated through March 2026 and incorporates a patient-level multinomial logistic regression analysis, showing that country remained strongly associated with maintenance strategy after adjustment for measured baseline clinical and disease characteristics.

Previous real-world studies provide important context, although most were conducted before the contemporary first-line maintenance era or included broader advanced ovarian cancer populations. In a chart-review study from the United States (US) and Europe, Hall et al. [17] described practice in 2017–2018, when first-line maintenance was predominantly bevacizumab-based. Among patients receiving first-line maintenance, 81% received bevacizumab and only 6% received a PARP inhibitor; use of targeted therapy also varied substantially by country, ranging from 70% to 99% among patients receiving maintenance. Audibert et al. [20] similarly showed that country-level variation was already present before widespread first-line PARP inhibitor use. In their oncologist survey, BRCA testing rates ranged from 45% in IT and 50% in the UK to 61% in FR, 65% in DE, and 73% in the US. Among patients with BRCA-negative disease, German physicians reported frequent use of bevacizumab with chemotherapy in the first-line setting, whereas platinum-based chemotherapy alone was more commonly reported in the other surveyed countries. More recent data suggest that PARP inhibitor adoption changed the maintenance landscape but did not make practice uniform. Moore et al. [16] analysed 7072 patient records from Europe and the US, including 5386 patients with stage III–IV ovarian cancer diagnosed between 2017 and 2020. Over time, BRCA and HRD testing increased, first-line maintenance PARP inhibitor monotherapy became more common, and anti-VEGF monotherapy decreased. However, treatment patterns continued to vary by country, indicating that the introduction of PARP inhibitors did not eliminate geographical differences in first-line treatment selection. In a more contemporary US analysis of 599 patients treated after 2020, Chase et al. [21] reported that only 48.2% received first-line maintenance therapy, with active surveillance used in 51.8%; among maintenance-treated patients, PARP inhibitor monotherapy was the most common strategy. Furthermore, a recent online physician survey [22] presented at ISPOR Europe 2024 provides a particularly relevant comparator, as it was conducted in the same five countries as the present study: FR, DE, IT, ES, and the UK. The survey included 3742 first-line ovarian cancer patients treated between January 2023 and April 2024. Bevacizumab remained the most commonly prescribed maintenance therapy, followed by niraparib and olaparib, and treatment patterns differed according to country, biomarker status, CA-125 level, radiographic progression, comorbidities, and ECOG performance status. Our study extends these observations by focusing on a more defined group of patients.

By excluding BRCA-mutated and HRD-positive disease, we focused on patients without a positive molecular result to support a biomarker-selected PARP inhibitor strategy. In this setting, the choice between bevacizumab continuation, PARP inhibitor maintenance, and active surveillance may be more dependent on treatment pathway, expected absolute benefit, toxicity, access, and reimbursement [9,16,17]. These factors may partly account for the distinct national patterns observed in our cohort: FR was characterised by high bevacizumab use, ES and the UK by high niraparib use, and the UK and IT by more frequent active surveillance. Formal access criteria may partly explain selected treatment patterns, although they do not account for all country-level variation.

European guidance recognises niraparib, bevacizumab and rucaparib as first-line maintenance options in selected patients with BRCA-wild-type or HRD-negative advanced epithelial ovarian cancer, whereas olaparib-based maintenance is primarily positioned for BRCA-mutated or HRD-positive disease [9]. The most informative differences related to national access criteria and to the timing of reimbursement or positioning for individual agents. The low use of bevacizumab in the UK is the clearest example. Under NHS England Cancer Drugs Fund criteria [23], first-line bevacizumab is restricted to higher-risk disease, including FIGO stage IV disease or selected FIGO stage III patients with residual disease, unsuitable for debulking surgery, or requiring neoadjuvant chemotherapy because optimal primary cytoreduction is considered unlikely. These criteria may partly explain the markedly lower bevacizumab use observed in the UK cohort. The high use of bevacizumab in FR appears to have a different basis. We did not identify a formal French reimbursement criterion that uniquely favours bevacizumab over niraparib in biomarker-negative disease. However, the French pattern is consistent with previous real-world evidence. Moore et al. [16] reported that, among patients receiving first-line maintenance in FR between 2017 and 2020, anti-VEGF monotherapy was particularly frequent and PARP inhibitor monotherapy comparatively uncommon. In addition, the French ENCOURAGE/GINECO study [24] documented enhanced real-world use of first-line bevacizumab-containing therapy in routine French practice before the contemporary PARP inhibitor era. The higher use of niraparib in ES may be compatible with favourable national positioning. Spanish regulatory sources identified relatively early niraparib as a recommended and reimbursed first-line maintenance option for patients without germline or somatic BRCA1/2 mutation and with HRD-negative disease after response to platinum-based chemotherapy [25,26]. In contrast, national assessments in DE and IT were relatively less favourable. In DE, the Federal Joint Committee (Gemeinsamer Bundesausschuss, G-BA) [27] concluded that an additional benefit of niraparib over the appropriate comparator therapy was not proven. Moreover, the Italian Medicines Agency (Agenzia Italiana del Farmaco, AIFA) [28] recognised conditional therapeutic innovation only for BRCA-mutated disease and not for BRCA wild-type disease.

The low use of rucaparib across countries is likely to reflect its later entry into the first-line maintenance landscape. The pivotal ATHENA-MONO trial [15] was published in 2022, approximately three years after the PRIMA trial [14] established niraparib in the same setting. A potential role of access and reimbursement is also suggested by a bevacizumab-focused analysis of 2017–2018 survey data from FR, DE, IT, ES, the UK, and the US. Overall, 82% of patients received chemotherapy alone and 18% received chemotherapy plus bevacizumab followed by bevacizumab maintenance. Bevacizumab use was higher in the US than in the European countries, and non-uniform treatment patterns were attributed in part to differences in approval timing and reimbursement [18]. Our findings suggest that access, reimbursement, and other country-level factors may remain relevant in the contemporary maintenance era and may contribute to differences in the balance between bevacizumab, PARP inhibitor maintenance, and surveillance among patients without an actionable BRCA/HRD biomarker.

This study has several strengths. It included a large contemporary cohort of patients treated across five European countries during a period in which bevacizumab, niraparib, rucaparib and active surveillance were all relevant first-line maintenance options. The analysis focused on a clinically important population of patients with BRCA wild-type, HRD-negative or biomarker-unknown disease, which remains under-represented in real-world maintenance studies [29,30]. In addition, the availability of patient-level data allowed adjusted analysis rather than relying only on aggregate country-level comparisons.

The study also has important limitations. The data were obtained from physician-reported patient charts rather than from a population-based registry and may not fully represent national prescribing rates. Although physicians were instructed to report their most recently seen eligible patients and not to preferentially select memorable cases, final patient selection remained at their discretion and was not strictly consecutive. Consequently, selection bias cannot be excluded and may have affected the observed treatment distributions. The analysis describes treatment allocation but does not include clinical outcomes, treatment duration, discontinuation, toxicity, dose modification or quality of life. 

## 5. Conclusions

In this contemporary five-country European real-world study of AEOC patients without an actionable BRCA/HRD biomarker, first-line maintenance treatment allocation varied substantially across FR, DE, IT, ES and the UK, and country remained strongly associated with maintenance strategy after adjustment for measured clinical factors. Some of the observed differences were probably consistent with national access criteria or reimbursement timing, particularly the low use of bevacizumab in the UK and the low use of rucaparib across all five countries. Other patterns, including the high use of bevacizumab in FR and niraparib in ES, appeared more consistent with established national treatment pathways, timing of drug positioning and clinical familiarity with specific agents. Future real-world studies should determine whether these differences in treatment allocation are associated with differences in PFS, OS, toxicity, treatment discontinuation and patient-reported outcomes. This will be particularly important for patients without an actionable BRCA/HRD biomarker, for whom the optimal maintenance strategy remains uncertain.

## Figures and Tables

**Table 1:** Baseline characteristics by country. Abbreviations: DE, Germany; ECOG, Eastern Cooperative Oncology Group; ES, Spain; FIGO, International Federation of Gynecology and Obstetrics; FR, France; HRD, homologous recombination deficiency; IT, Italy; and UK, United Kingdom.

Characteristic	FR N = 751 No. (%)	DE N = 589 No. (%)	IT N = 633 No. (%)	ES N = 618 No. (%)	UK N = 702 No. (%)	Overall N = 3293 No. (%)
**Age group, years**						
<40	9 (1.2)	10 (1.7)	5 (0.8)	1 (0.2)	5 (0.7)	30 (0.9)
40–59	125 (16.6)	146 (24.8)	90 (14.2)	167 (27.0)	148 (21.1)	676 (20.5)
60–69	316 (42.1)	247 (41.9)	342 (54.0)	270 (43.7)	333 (47.4)	1508 (45.8)
70–79	248 (33.0)	161 (27.3)	169 (26.7)	160 (25.9)	193 (27.5)	931 (28.3)
≥80	53 (7.1)	25 (4.2)	27 (4.3)	20 (3.2)	23 (3.3)	148 (4.5)
**FIGO stage**						
III	381 (50.7)	339 (57.6)	163 (25.8)	290 (46.9)	277 (39.5)	1450 (44.0)
IV	370 (49.3)	250 (42.4)	470 (74.2)	328 (53.1)	425 (60.5)	1843 (56.0)
**ECOG performance status**						
0	88 (11.7)	138 (23.4)	177 (28.0)	117 (18.9)	116 (16.5)	636 (19.3)
1	537 (71.5)	268 (45.5)	373 (58.9)	445 (72.0)	518 (73.8)	2141 (65.0)
≥2	126 (16.8)	183 (31.1)	83 (13.1)	56 (9.1)	68 (9.7)	516 (15.7)
**Histology**						
High-grade serous	682 (90.8)	384 (65.2)	492 (77.7)	515 (83.3)	562 (80.1)	2635 (80.0)
Other	69 (9.2)	205 (34.8)	141 (22.3)	103 (16.7)	140 (19.9)	658 (20.0)
**Biomarker subgroup**						
BRCA wild-type/HRD-negative	686 (91.3)	503 (85.4)	555 (87.7)	577 (93.4)	630 (89.7)	2951 (89.6)
BRCA wild-type/HRD unknown/untested	33 (4.4)	23 (3.9)	41 (6.5)	27 (4.4)	39 (5.6)	163 (4.9)
Biomarker unknown/untested	32 (4.3)	63 (10.7)	37 (5.8)	14 (2.3)	33 (4.7)	179 (5.4)
**Response to induction therapy**						
Complete response	423 (56.3)	299 (50.8)	238 (37.6)	263 (42.6)	325 (46.3)	1548 (47.0)
Partial response	318 (42.3)	285 (48.4)	370 (58.5)	345 (55.8)	361 (51.4)	1679 (51.0)
Stable disease	10 (1.3)	5 (0.8)	25 (3.9)	10 (1.6)	16 (2.3)	66 (2.0)
**Bevacizumab use during induction**						
Yes	323 (43.0)	250 (42.4)	266 (42.0)	214 (34.6)	136 (19.4)	1189 (36.1)
No	428 (57.0)	339 (57.6)	367 (58.0)	404 (65.4)	566 (80.6)	2104 (63.9)
**Surgical outcome**						
No debulking surgery	292 (38.9)	140 (23.8)	257 (40.6)	209 (33.8)	247 (35.2)	1145 (34.8)
Surgery with no residual disease	234 (31.2)	162 (27.5)	136 (21.5)	177 (28.6)	233 (33.2)	942 (28.6)
Surgery with optimal cytoreduction	180 (24.0)	203 (34.5)	155 (24.5)	154 (24.9)	172 (24.5)	864 (26.2)
Surgery with suboptimal cytoreduction	45 (6.0)	84 (14.3)	85 (13.4)	78 (12.6)	50 (7.1)	342 (10.4)

**Table 2:** Standardised residuals for first-line maintenance allocation by country. The table shows country–treatment combinations with absolute adjusted standardised residuals ≥ 6.0. Positive values indicate higher-than-expected use and negative values lower-than-expected use under the null hypothesis of no association between country and maintenance strategy. The overall chi-square test for maintenance allocation by country was significant (chi-square = 725.4, df = 16, *p* < 0.001). Abbreviations: DE, Germany; ES, Spain; FR, France; IT, Italy; and UK, United Kingdom.

Country	Maintenance Strategy	Observed	Expected	Standardised Residual
FR	Bevacizumab	477	295.3	15.45
UK	Bevacizumab	124	276.1	−13.25
DE	Other	124	49.4	12.25
ES	Niraparib	316	204.0	10.63
UK	Surveillance	185	101.7	10.07
FR	Niraparib	142	247.9	−9.35
IT	Surveillance	157	91.7	8.21
FR	Surveillance	40	108.8	−8.12
UK	Niraparib	308	231.7	6.90

**Table 3:** Likelihood-ratio tests from the multinomial logistic regression model. Likelihood-ratio tests compared the full multinomial logistic regression model with reduced models omitting each variable in turn. The outcome was maintenance strategy, categorised as bevacizumab, PARP inhibitor maintenance, or active surveillance. Abbreviations: CR, complete response; ECOG, Eastern Cooperative Oncology Group; FIGO, International Federation of Gynecology and Obstetrics; HG, high-grade; HRD, homologous recombination deficiency; LR, likelihood ratio; neg, negative; R0, no residual disease; R1, optimal cytoreduction; R2, suboptimal cytoreduction; and wt, wild-type.

Variable	LR χ^2^	df	*p* Value
**Country**	550.1	8	<0.001
Surgical outcome No surgery/R0/R1/R2	81.6	6	<0.001
Age <70 vs. ≥70 years	29.2	2	<0.001
ECOG performance status 0/1/≥2	18.7	4	<0.001
Response to induction therapy CR vs. non-CR	13.0	2	0.0015
Histology HG serous vs. other	8.7	2	0.013
Biomarker subgroup BRCA wt/HRD neg/BRCA wt/HRD unknown/biomarker unknown	4.4	4	0.356
FIGO stage III vs. IV	2.6	2	0.272

## Data Availability

The original contributions presented in this study are included in the article. Further inquiries can be directed to the corresponding author.

## Supplementary Materials

The following supporting information can be downloaded at: https://www.mdpi.com/article/10.3390/cancers18162682/s1, Table S1: Complete multinomial logistic regression results for first-line maintenance strategy (N = 3017).
