# Supplementary material for: First-Line Maintenance Therapy Patterns in BRCA Wild-Type, HRD-Negative or Biomarker-Unknown Advanced Epithelial Ovarian Cancer: A Five-Country European Real-World Study by APLUSA
Source: Cancers (Basel). 2026 Aug 19;18(16):2682. doi: 10.3390/cancers18162682 (PMC13511211; doi:10.3390/cancers18162682)
Supplement: Supplementary file 1 [file cancers-18-02682-s001.zip › cancers-4452503-supplementary.pdf]

Table S1. Complete multinomial logistic regression results for first-line maintenance strategy (N=3017).

Predictor (comparison)	Bevacizumab vs active surveillance			PARP inhibitor vs active surveillance		
	β	OR (95% CI)	P value	β	OR (95% CI)	P value
**Country:** *France vs UK*	3.02	20.55 (13.64-30.96)	<0.001	0.83	2.29 (1.53-3.42)	<0.001
**Country:** *Germany vs UK*	2.16	8.68 (5.75-13.11)	<0.001	0.89	2.43 (1.64-3.58)	<0.001
**Country:** *Italy vs UK*	0.77	2.17 (1.58-2.98)	<0.001	-0.52	0.59 (0.45-0.80)	<0.001
**Country:** *Spain vs UK*	1.80	6.06 (4.08-9.01)	<0.001	1.31	3.69 (2.58-5.27)	<0.001
**Surgical outcome:** *R0 vs no surgery*	-1.14	0.32 (0.23-0.45)	<0.001	-0.88	0.42 (0.30-0.58)	<0.001
**Surgical outcome:** *R1 vs no surgery*	-0.39	0.68 (0.49-0.95)	0.02	-0.23	0.79 (0.58-1.10)	0.16
**Surgical outcome:** *R2 vs no surgery*	0.98	2.67 (1.56-4.55)	<0.001	0.84	2.32 (1.36-3.96)	0.002
**Age:** *≥70 vs <70 yr*	-0.69	0.50 (0.39-0.64)	<0.001	-0.49	0.61 (0.48-0.78)	<0.001
**ECOG performance status:** *1 vs 0*	-0.05	0.95 (0.70-1.29)	0.75	-0.07	0.93 (0.69-1.25)	0.63
**ECOG performance status:** *≥2 vs 0*	-0.74	0.48 (0.31-0.72)	<0.001	-0.72	0.49 (0.32-0.73)	<0.001
**Response to induction therapy:** *complete vs noncomplete*	0.46	1.59 (1.21-2.08)	<0.001	0.44	1.55 (1.19-2.01)	<0.001
**Histology:** *high-grade serous vs other*	0.19	1.21 (0.91-1.63)	0.19	0.41	1.50 (1.13-2.00)	0.005
**Biomarker status:** *BRCA wt/HRD unk vs BRCA wt/HRD-neg*	-0.18	0.83 (0.49-1.41)	0.50	0.06	1.06 (0.65-1.74)	0.81
**Biomarker status:** *Biomarker unk vs BRCA wt/HRD-neg*	0.19	1.21 (0.67-2.19)	0.53	0.45	1.56 (0.88-2.78)	0.13
**FIGO stage:** *IV vs III*	-0.07	0.93 (0.71-1.23)	0.62	0.09	1.10 (0.85-1.42)	0.48

The model compared bevacizumab maintenance and PARP inhibitor maintenance with active surveillance. Active surveillance was the reference outcome, and all predictors were entered simultaneously. β denotes the multinomial log-odds coefficient. P values are two-sided Wald P values. Abbreviations: CI, confidence interval; ECOG, Eastern Cooperative Oncology Group; FIGO, International Federation of Gynecology and Obstetrics; HRD, homologous recombination deficiency; neg, negative; OR, odds ratio; PARP, poly(ADP-ribose) polymerase; R0, no residual disease; R1, optimal cytoreduction; R2, suboptimal cytoreduction; UK, United Kingdom; unk, unknown/untested; wt, wild-type.
